# Large retroperitoneal extraskeletal Ewing’s sarcoma with renal pedicle invasion: a case report

**DOI:** 10.1186/s12894-023-01272-z

**Published:** 2023-05-16

**Authors:** Shu-Yu Wu, Chun-Kai Hsu, Chung-Tai Yue, Yao-Chou Tsai

**Affiliations:** 1 Department of Urology, Taipei Tzu Chi Hospital, Buddhist Tzu Chi Medical Foundation, No. 289, Jianguo Rd., Xindian Dist, New Taipei City, 231 Taiwan; 2 Department of Urology, School of medicine, Tzu Chi University, Hualien, Taiwan; 3 Department of Pathology, Taipei Tzu Chi Hospital, Buddhist Tzu Chi Medical Foundation, New Taipei City, Taiwan

**Keywords:** Retroperitoneal tumor, Extraskeletal Ewing’s sarcoma, Transarterial embolization

## Abstract

**Background:**

Extraskeletal Ewing’s sarcoma (EES) is a rare malignant tumor primarily found in children and young adults. Localized disease can present with nonspecific symptoms such as local mass, regional pain, and increased skin temperature. More severe cases may present with systemic symptoms such as malaise, weakness, fever, anemia, and weight loss. Among these lesions, retroperitoneal sarcomas are relatively uncommon and difficult to diagnose. Since they are usually asymptomatic until large enough to compress or invade the surrounding tissues, most are already advanced at first detection. Traditionally, the treatment of choice is complete surgical resection, sometimes combined with postoperative radiotherapy and chemotherapy. We report a case of EES with left renal artery invasion in the left retroperitoneal cavity successfully treated with transarterial embolization and surgery.

**Case presentation:**

A 57-year-old woman with a negative family history of cancer presented at our Urology Department with a large left retroperitoneal tumor found by magnetic resonance imaging during the health exam. Physical examination showed a soft abdomen and no palpable mass or tenderness. Imaging studies showed that the tumor covered the entire left renal pedicle, but the left kidney, left adrenal gland, and pancreas appeared tumor free. Since the tumor tightly covered the entire renal pedicle, tumor excision with radical nephrectomy was advised. The patient underwent transarterial embolization of the left renal artery with 10 mg of Gelfoam pieces daily before surgical excision. Tumor excision and left radical nephrectomy were uneventful the day after embolization. Post-operatively, the patient recovered well and was discharged on day 10. The final histopathological analysis showed a round blue cell tumor consistent with an Ewing sarcoma, and the surgical margins were tumor free.

**Conclusions:**

Retroperitoneal malignancies are rare but usually severe conditions. Our case report showed that retroperitoneal EES with renal artery invasion could be treated safely with transarterial embolization and surgery.

## Background

Ewing’s sarcoma (ES) is a rare malignant tumor composed of small round cells, which Ewing first described in 1921 [1]. ESs have been reported throughout the human body. They can be classified as ES of the bone, extraskeletal ES (EES), malignant small cell tumors of the chest wall (Askin tumor), and soft tissue-based primitive neuroectodermal tumors (PNET) [2, 3]. EESs most often occur in soft tissues in the paravertebral region, chest, extremities, and retroperitoneal [4]. Most cases involve children, adolescents, and young adults, with > 90% of cases having disease onset between the ages of four and 25 [5]. Local masses, localized regional pain with variable intensity, increased skin temperature, and restricted limb movement due to nerve invasion are common [6]. Systemic symptoms such as malaise, weakness, fever, anemia, and weight loss may occur in metastasis disease [4]. Unfortunately, these symptoms are nonspecific and cannot distinguish EES from other tumors. Traditionally, treatment of choice is complete surgical resection, sometimes combined with postoperative radiotherapy and chemotherapy [7, 8]. Even after complete therapy, patients with EES have poor prognoses and high metastasis or recurrence risks [9].

Retroperitoneal sarcoma is relatively uncommon, accounting for < 15% of all soft tissue sarcomas [10]. They are usually asymptomatic until large enough to compress or invade the surrounding tissues. Since patients often come to medical attention with an incidentally observed mass lesion found during a health exam or image studies for other reasons, most tumors are already advanced at first detection [11]. Patients with high-grade, rapidly expanding tumors may present with fevers and leukocytosis. Laboratory examinations are usually uninformative in diagnosing these tumors [12]. Radiographic imaging is a key component of evaluating patients with retroperitoneal tumors. Contrast-enhanced computed tomography (CT) is often the preferred tool for detecting the primary retroperitoneal lesion [13]. Magnetic resonance imaging (MRI) with gadolinium is an alternative for patients with allergies to iodinated contrast agents. It is superior for delineating the tumor’s extension into the surrounding tissues [14]. Due to their nonspecific clinical characteristics, retroperitoneal EESs often have delayed diagnosis and treatment. Here, we report a case of EES with left renal artery invasion in the left retroperitoneal cavity successfully treated with transarterial embolization and surgery.

## Case presentation

**Fig. 1:**
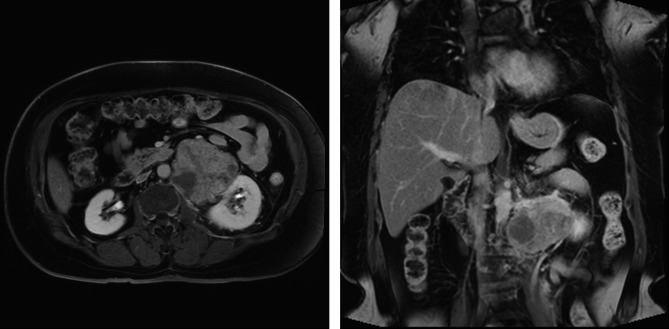
The MRI showed a heterogeneous left retroperitoneal tumor

**Fig. 2:**
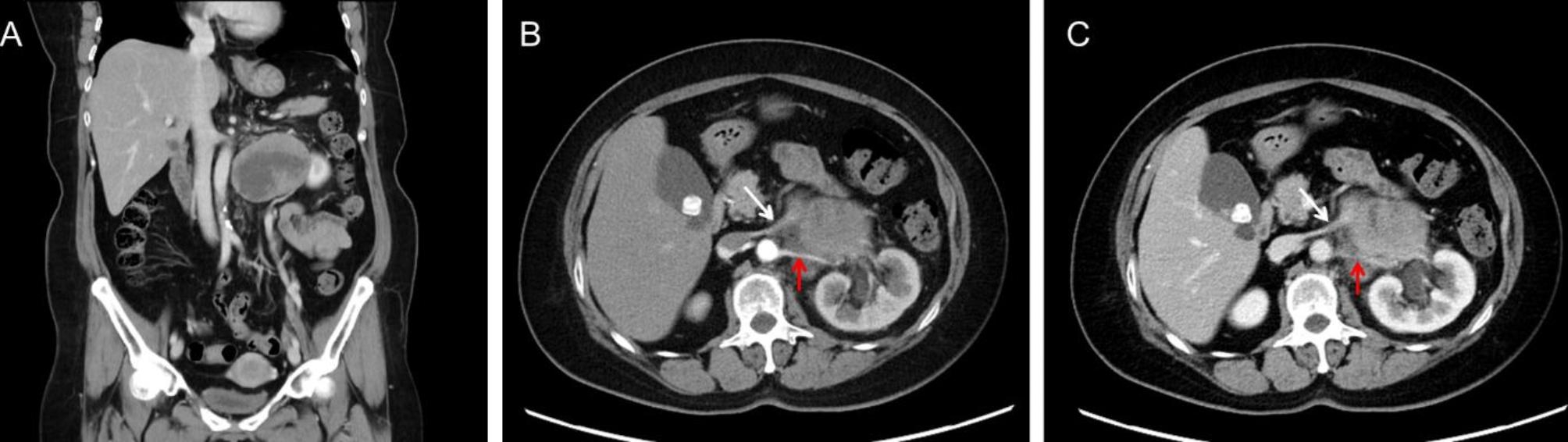
The CT showed a left para-aortic heterogeneous tumor with a mass effect on the left kidney (A) that encircles the left renal pedicle: (B) Arterial phase; (C) Venous phase. The left renal artery (red arrow) and vein (white arrow) are indicated

A 57-year-old woman with a negative family history of cancer presented at our Urology Department with a large left retroperitoneal tumor found by MRI during the health exam (Fig. 1). Physical examination showed a soft abdomen and no palpable mass or tenderness. An abdominal CT scan showed an 8.3 × 8.5 × 6.8 cm heterogeneous tumor in the left para-aortic region with a mass effect on the left kidney (Fig. 2A). Imaging studies showed the tumor covered the entire left renal pedicle, but the left kidney, left adrenal gland, and pancreas appeared free of tumor. The tentative diagnosis was retroperitoneal sarcoma or liposarcoma. Therefore, surgical excision was indicated.

**Fig. 3:**
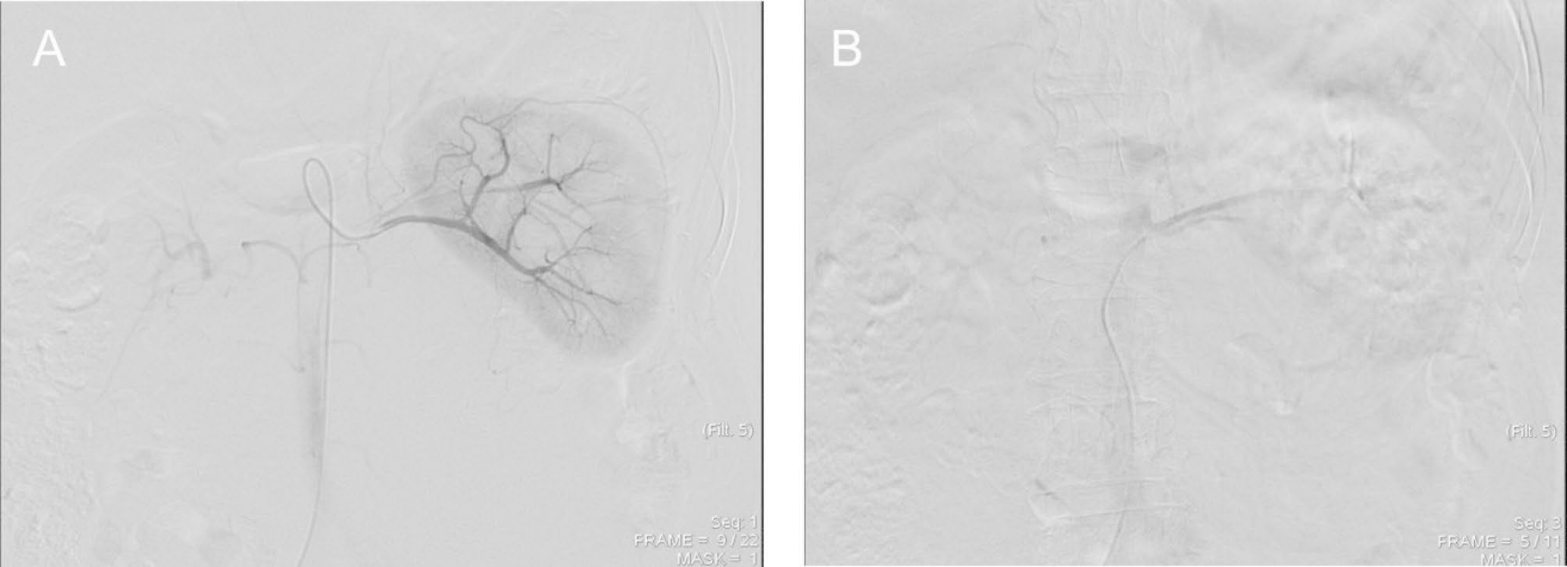
Transarterial embolization of the left renal artery via the right femoral artery. (A) Before embolization. (B) After embolization

The patient consented to an exploratory laparotomy with tumor resection and left radical nephrectomy. Since the tumor tightly covered the entire renal pedicle (Fig. 2B-C), tumor excision with radical nephrectomy was advised. Consider renal artery control and ligation prior to ligation of the renal veins may not be feasible in this special case. Besides, significant collateral feeding vessels to this perivascular tumor might hamper the pedicle control and very likely increase risk of intraoperative bleeding. To reduce the bleeding risk and possible intra-operative complications, the patient received a single transarterial embolization of the left renal artery with 10 mg of Gelfoam pieces daily before surgical excision (Fig. 3). The operation began with a midline incision, and exploration revealed a larger retroperitoneal mass invading the left renal pedicle that was tightly adhered and could not be separated. No direct aortic invasion was noted after careful tumor and aorta dissection. Since the left renal artery had been embolized, proper pedicle control was easily achieved with suture ligation. The entire tumor could be easily removed with adequate control of its blood supply. There was some adhesion between the tumor and the psoas muscle, which was negative for malignancy. Hemostasis was maintained, and the abdomen and skin were closed in the usual manner.

**Fig. 4:**
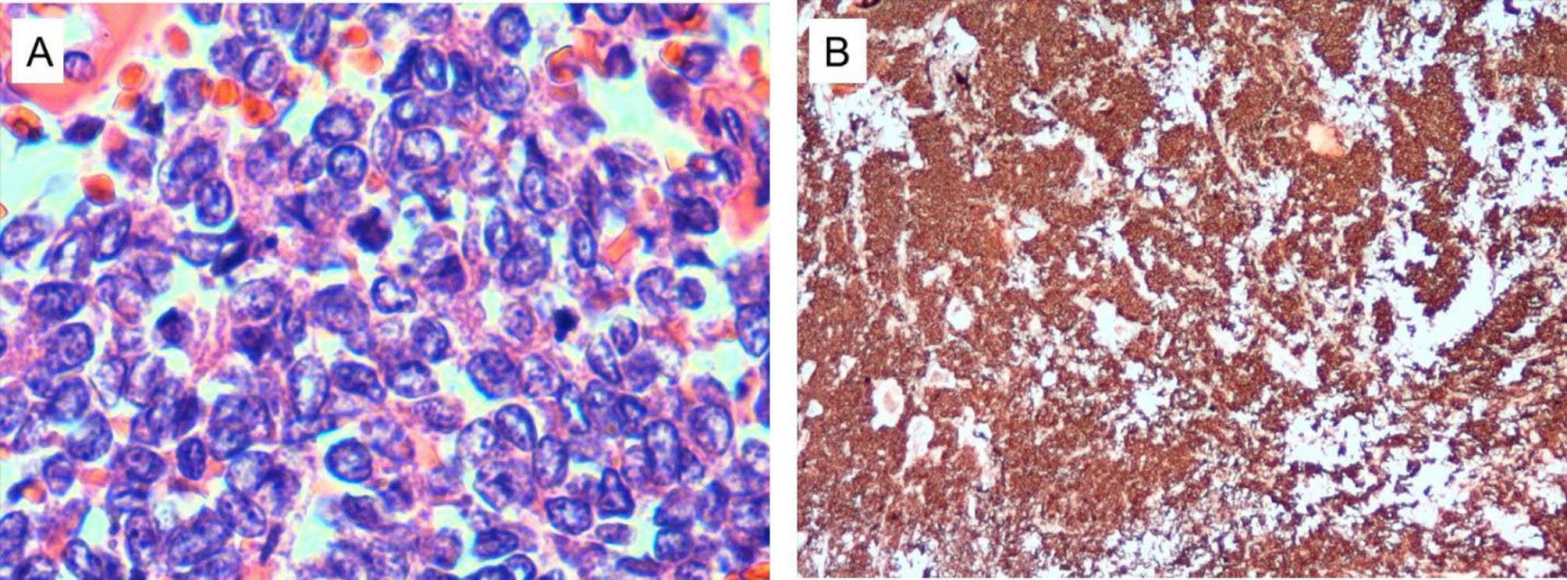
(A) Hematoxylin and eosin staining showed tumor cells with round nuclei and pale cytoplasm (400× magnification). (B) Immunohistochemistry showed CD99-positive tumor cells (20× magnification)

Post-operatively, the patient recovered well and was discharged on day 10. The final histopathological analysis showed a round blue cell tumor consistent with ES/PNET (Fig. 4). Fortunately, the surgical margins were tumor free, so the resection was considered complete. Adjuvant chemotherapy and radiotherapy were to be performed later by an oncologist.

The postoperative follow-up showed that the patient recovered well. The most recent clinic follow-up so far was 6 months after the operation, and the imaging studies showed no tumor recurrence. During this period, the patient has received complete radiation therapy (44 Gy/22fx, tumor bed and left kidney region) and is receiving chemotherapy (vincristine, doxorubicin, and cyclophosphamide). No serious adverse effects other than mild leukopenia. The patient is still under follow-up and treatment in our hospital.

## Discussion and conclusions

Most retroperitoneal tumors are malignant and account for one-third of soft tissue sarcomas. They usually present as large masses at primary diagnosis. Patients will be asymptomatic until the mass is large enough to compress or invade contagious structures. Patients may have nonspecific abdominal symptoms such as abdominal discomfort, distention, nausea, and vomiting [15, 16]. The family of ES and related tumors is characterized by small round blue cell tumors associated with a nonrandom t(11:22)(q24:q12) chromosome rearrangement [4, 17]. ESs and PNETs are well-known tumors in this family. They are malignant small blue round cell tumors with variable degrees of neuroectodermal differentiation [18]. Since the most common site of ES is the bone, most pediatric patients have so-called skeletal ESs, while ESS is very rare. Unlike children, more than 50% of ESs in adults are ESS, which can develop in the trunk, intraabdominal tissues, retroperitoneum, and viscera [18–20]. These tumors often present with rapid growth and widespread metastasis, leading to poor prognoses [21].

Imaging studies are essential for diagnosing such retroperitoneal lesions. MRI is the preferred tool for delineating the tumor’s extent and relationship with adjacent tissues or blood supplies [16]. However, while imaging studies are necessary, they cannot make a definite diagnosis due to other equivalent tumors having the same imaging characteristics [18]. Tissue confirmation of such tumors is always required. Specific stains are helpful for diagnosis, including those for the CD99 molecule (Xg blood group), micrometastases, vimentin, nonspecific esterase, S100 calcium-binding proteins, desmin, and cytokeratins [20–23]. Multimodal treatment consisting of surgical resection, chemotherapy, and high-dose radiotherapy (if indicated) has been recommended [24]. Prognostic factors are similar for ESS and ES, such as the presence or absence of metastasis, tumor size, extent of necrosis, and response to chemotherapy [25]. The quality of the primary excision is also important for local and distant recurrence, and wider resection margins are required [26]. Furthermore, combining surgery with chemotherapy and/or radiotherapy is recommended based on the location, respectability, and tumor stage [27]. The patients should undergo radiotherapy when negative surgical margins cannot be achieved [8]. In patients with distant metastasis, chemotherapy remains an option after primary tumor excision, providing better progression-free survival [28]. In some reports, targeted therapy and immunotherapy also play a role in ES treatment [29, 30]. To our knowledge, this is the first case report of ESS presenting with renal artery invasion but a normal kidney. In our case, the patient had nonmetastatic disease. She received a tumor resection with negative surgical margins after renal artery embolization. She underwent chemotherapy and radiotherapy to avoid local and distant metastasis. Continuous follow-up is necessary for treatment outcome evaluations.

In conclusion, retroperitoneal malignancies are rare but usually severe conditions. Their clinical symptoms are usually nonspecific. Imaging studies are required for primary diagnosis, but a definite diagnosis still depends on histopathology. EES is one of the differential diagnoses of retroperitoneal malignancy, characterized by small blue round cells. Multimodality treatment comprising surgical resection, chemotherapy, and radiotherapy is recommended for better outcomes. Our case report shows that retroperitoneal EESs with renal artery invasion can be treated safely with transarterial embolization and surgery.

## Data Availability

The datasets used and/or analyzed during the current study available from the corresponding author on reasonable request.

## List of Abbreviations

ES: Ewing’s sarcoma
EES: Extraskeletal Ewing’s sarcoma
PNET: Primitive neuroectodermal tumors
CT: Computed tomography
MRI: Magnetic resonance imaging
